# MUSTANG-MR Structural Sieving Server: Applications in Protein Structural Analysis and Crystallography

**DOI:** 10.1371/journal.pone.0010048

**Published:** 2010-04-06

**Authors:** Arun S. Konagurthu, Cyril F. Reboul, Jason W. Schmidberger, James A. Irving, Arthur M. Lesk, Peter J. Stuckey, James C. Whisstock, Ashley M. Buckle

**Affiliations:** 1 NICTA Victoria Research Laboratory at The University of Melbourne, The University of Melbourne, Melbourne, Australia; 2 Department of Computer Science and Software Engineering, The University of Melbourne, Victoria, Australia; 3 Department of Biochemistry and Molecular Biology, Monash University, Victoria, Australia; 4 ARC Centre of Excellence in Structural and Functional Microbial Genomics, Monash University, Victoria, Australia; 5 The Huck Institute for Genomics, Proteomics, and Bioinformatics, Department of Biochemistry and Molecular Biology, Pennsylvania State University, University Park, Pennsylvania, United States of America; Leeds Institute of Molecular Medicine, United Kingdom

## Abstract

**Background:**

A central tenet of structural biology is that related proteins of common function share structural similarity. This has key practical consequences for the derivation and analysis of protein structures, and is exploited by the process of “molecular sieving” whereby a common core is progressively distilled from a comparison of two or more protein structures. This paper reports a novel web server for “sieving” of protein structures, based on the multiple structural alignment program MUSTANG.

**Methodology/Principal Findings:**

“Sieved” models are generated from MUSTANG-generated multiple alignment and superpositions by iteratively filtering out noisy residue-residue correspondences, until the resultant correspondences in the models are optimally “superposable” under a threshold of RMSD. This residue-level sieving is also accompanied by iterative elimination of the poorly fitting structures from the input ensemble. Therefore, by varying the thresholds of RMSD and the cardinality of the ensemble, multiple sieved models are generated for a given multiple alignment and superposition from MUSTANG. To aid the identification of structurally conserved regions of functional importance in an ensemble of protein structures, Lesk-Hubbard graphs are generated, plotting the number of residue correspondences in a superposition as a function of its corresponding RMSD. The conserved “core” (or typically active site) shows a linear trend, which becomes exponential as divergent parts of the structure are included into the superposition.

**Conclusions:**

The application addresses two fundamental problems in structural biology: First, the identification of common substructures among structurally related proteins—an important problem in characterization and prediction of function; second, generation of sieved models with demonstrated uses in protein crystallographic structure determination using the technique of Molecular Replacement.

## Introduction

### Prediction of protein function

Understanding structural similarity between proteins within a homologous family as well as between distant or even unrelated proteins is a common task in molecular biology. In addition, the prediction of protein function remains a serious challenge, but the observation that the active sites of proteins are often the best preserved regions in a divergent family offers a robust method of classifying proteins of unknown function by structural alignment. This has been exploited by a “sieving” procedure that iteratively identifies matching residues in a multiple structural alignment that fit below a threshold root-mean-square-deviation (RMSD [1], [2]). Examination of residues remaining after sieving allows the identification of functional residues in proteins of unknown function.

### Sieved models in crystal structure determination

The most common method of protein structure determination is molecular replacement (MR). This technique involves using the structure of a protein that shares significant sequence similarity with the protein of unknown structure as a starting point in the structure determination (otherwise known as solving the phase problem). The process generally involves four steps: (1) Using sequence-based searching methods such as PSI-BLAST [3] to identify suitable structures that can be used for MR; (2) modification of structures to yield search models; (3) Finding the orientation and position of the search model in the unit cell of the target crystal; (4) Refinement of the model.

Where the sequence similarity between the unknown target and the search model is high (sequence identity >40%) the success rate of MR is very good, even without optimisation of the search model. However, in cases where sequence similarity is low (identity <30%) MR, and subsequent structure refinement becomes non-trivial, and emphasis must be placed on the optimisation of the search model. Here, MR solutions are commonly challenging to refine (the so called “model bias” trap). This situation occurs where errors in regions of the starting model cannot be adequately identified and corrected due to model bias. However it is possible to remove model bias by removing regions of the structure that are predicted to be different in the search model and target, typically loops. However, this process is a subjective one and relies on sequence alignments, which are often incorrect, particularly at low sequence identity. Thus it is often unclear which loops should be removed and how much of the loop should be removed, and each model must be tested.

Sieving presents a robust solution to this method, because it produces search models with structurally divergent regions removed in an objective fashion. The ideal starting model (e.g. one with least model bias) is difficult to obtain *a priori*, however it is possible to test multiple sieved models and assess the refinement process using statistically robust validation, providing a generally applicable method for model bias reduction.

### Standardized structural comparisons

RMSD values are often used as a measure of structural similarity between homologous proteins, however a reported value will deviate considerably as a function of the number of residues considered. Hence, the resulting values may vary with the alignment program used, and/or the choice of parameters active in the calculation. The curves generated by the sieving procedure can be used to report multiple comparisons at a common threshold: the number of residues aligned at a specified RMSD, as a measure of the extent of residue-residue correspondence; or the RMSD at a specified number of aligned residues, representing the average extent of residue-residue deviation squared.

In this work we describe a novel web server for “sieving” of protein structures, based on the multiple structural alignment program MUSTANG. We show how this application can be used to produce standardized structural comparisons, identify common substructures among structurally related proteins, as well as generate sieved models for use in protein crystallographic structure determination using the technique of Molecular Replacement.

## Methods

MUSTANG-MR web server benefits from an intuitive interface to MUSTANG [4] and a specially designed sieving procedure. “Sieved” models are produced from MUSTANG-generated multiple structural alignment (MSA) and superpositions by iteratively filtering out noisy residue-residue correspondences, until the resultant correspondences in the models are optimally ‘superposable’ under a threshold of RMSD. This residue-level sieving is also accompanied by iterative elimination of the poorly fitting structures from the input ensemble. Therefore, by varying the thresholds of RMSD and the cardinality of the ensemble, multiple sieved models are generated for a given multiple alignment and superposition from MUSTANG.

**Figure 1:**
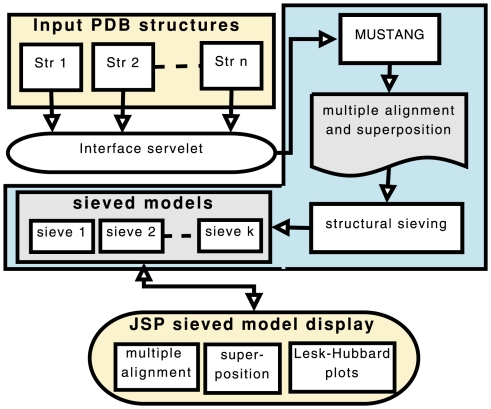
Overview of MUSTANG structural sieving server.

**Figure 2:**
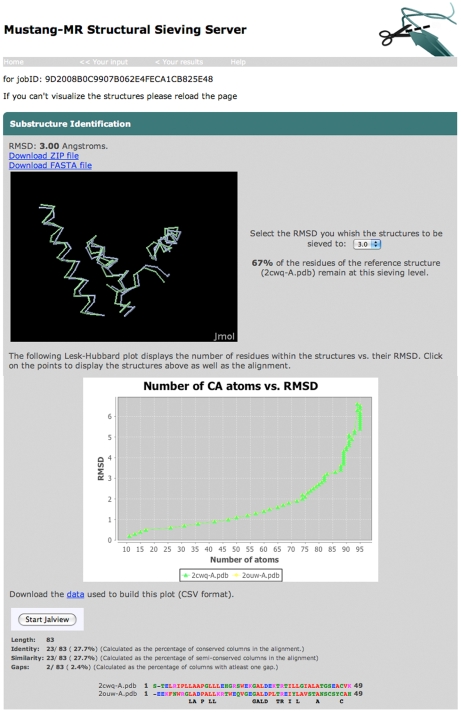
Screen snapshot of the results of a typical structural alignment, showing the sieving results in a Jmol window, Lesk-Hubbard plot, and structure-based sequence alignment.

MUSTANG-MR is a project fork on MUSTANG with sieving incorporated into it. MUSTANG-MR is implemented in C++ and is available under an open-source license. A user-friendly web interface is written in JSP/Java. Figure 1 illustrates the working of the sieving server. Multiple PDB files can be uploaded and processed rapidly. The server through its interface allows direct visual analysis of the results by giving access to a range of automated tools (Figure 2). Exhaustively sieved models can be displayed interactively. Graphical representations of alignments and superpositions of sieved structures are provided using Jmol (www.jmol.org), Jalview [5] and BioJava [6]. Superimposed structural coordinates and residue-level alignment can be accessed in PDB and FASTA formats respectively.

**Figure 3:**
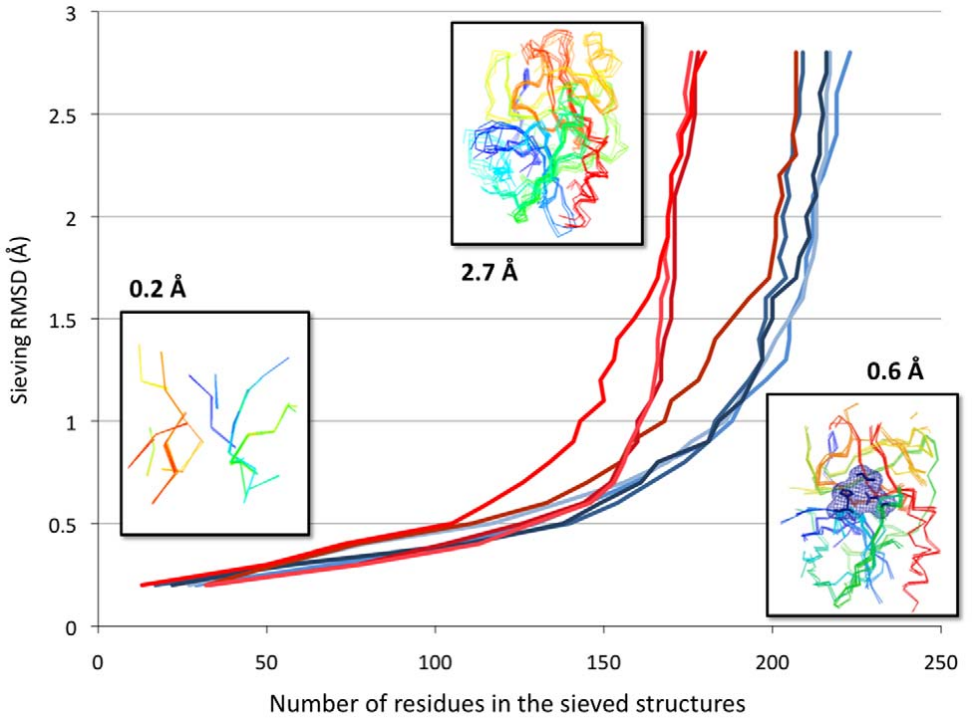
Plot of number of residue correspondences vs. RMSD in each structure. Eukaryotic proteases (3EST, 1TON, 3RP2, 5CHA) are in blue, prokaryotic (1SGT, 2SGA, 3SGB, 2ALP) in red. The boxes highlight superpositions of sieved structures with their corresponding RMSD. At 0.6 Å, the catalytic triad and its wireframe surface are displayed in dark blue.

To aid the identification of structurally conserved regions of functional importance in an ensemble of protein structures, Lesk-Hubbard graphs [1] are generated, plotting the number of residue correspondences (NCORE) in a superposition as a function of its corresponding RMSD (Figure 3). The conserved “core” (or typically active site) shows a linear trend, which becomes exponential as divergent parts of the structure are included into the superposition.

## Results and Discussion

### Substructure identification

A data set of 8 trypsin-like serine proteases (see Table 1) from 2 different SCOP [7] families was used to demonstrate the utility of the server for identification of structurally conserved “core”. MUSTANG generated alignment and superposition (NCORE = 143; RMSD = 2.7 Å) were sieved by varying RMSD thresholds from 0.2 Å to 2.7 Å. Figure 3 shows the Lesk-Hubbard plot for the serine proteases data set. In the part showing the exponential, the eukaryotic enzymes share an almost identical curve, while the prokaryotic enzymes behave in a heterogeneous fashion. This reflects a more extensive ‘superposable’ region amongst the eukaryotic proteins when compared to the prokaryotic enzymes, and greater structural diversity amongst the prokaryotic proteins outside the core region. Using the interactive components of the sieving tool, the semi-linear regions of these plots (0.2 Å to 0.8 Å) are found to correspond with a shared structural core between the two families. The catalytic triad His-Asp-Ser, typical of serine proteases, is present in the identified core above the sieving RMSD of 0.6 Å, considerably below the MUSTANG alignment and superposition RMSD.

**Table 1:** Details of Serine proteases used in structural alignment and sieving.

	SCOP Family	Name and species	PDB ID
Trypsin-like serine proteases	Eukaryotic Proteases	Porcine pancreatic elastase (*sus scrofa*)	3EST
		Tonin (*rattus rattus*)	1TON
		Mast cell protease II (*rattus rattus*)	3RP2
		α-Chymotrypsin A (*bos taurus*)	5CHA
	Prokaryotic Proteases	Trypsin (*streptomyces griseus*)	1SGT
		Protease I (*Achromobacter lyticus*)	1ARB
		Proteinase B (*Streptomyces griseus*)	3SGB
		α-Lytic protease (*Lysobacter enzymogenes*)	2ALP

Altogether this demonstrates that the functional residues of different families can be found within the best-conserved “cores”, and underlines the value of the “sieving” approach to substructure identification.

**Figure 4:**
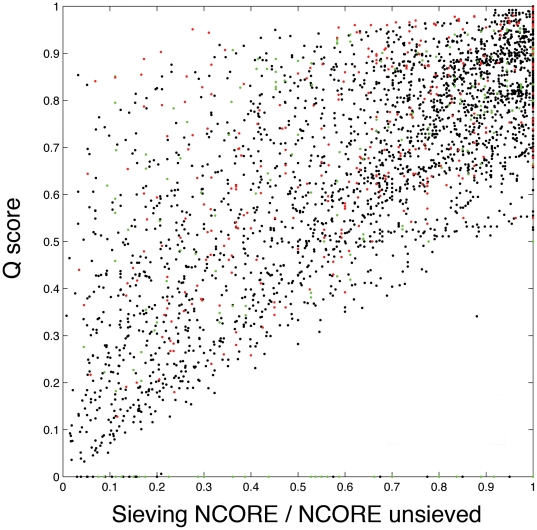
Performance of ‘sieving’ against manually curated alignments from SISYPHUS [8]. Q scores were computed using the program QSCORE. Colors correspond to the 3 category groups in the SISYPHUS database: Homologous (black), Fold (green) and Fragments (red). We did not observe any clear relationship between Q scores, sieving level and the three classes.

To further assess the validity of the sieving method we have performed an exhaustive benchmarking against the SISYPHUS database, which contains a set of 149 manually curated MSAs classified into 3 categories [8]. Comparison of MUSTANG-MR and SISYPHUS MSAs were performed by computing the PREFAB Q score [9]. SISYPHUS alignments were used as the reference in our comparisons, whose results are presented in figure 4. The ordinate gives the ‘Q score’ (or proportion of correctly aligned residue pairs) between SISYPHUS and ‘sieved’ alignments. The abscissa gives the value ‘NCORE/NCORE unsieved’ for all MSAs (at all sieving levels), where ‘NCORE’ is the number of aligned columns within an MSA at a sieving RMSD threshold, and ‘NCORE unsieved’ the number of aligned columns obtained from the initial MUSTANG alignment (prior to the start of sieving). The fraction ‘NCORE/NCORE unsieved’ has the advantage of providing a standardized representation for all the alignments and ranges between maximum sieving (i.e with low RMSD threshold) to the minimum sieving (i.e with high RMSD threshold). From figure 4, one can observe that a vast majority of points lie above and around the y = x diagonal, showing the method is able to identify an increasing number of relevant residue pairs as the number of residues taken into account increases. It should be noted that in most cases MUSTANG-MR alignment at some sieving level would yield lower Q scores than the unsieved (initial) alignment when compared with SISYPHUS benchmark. This is because the residue-residue alignments at various sieving levels have an incomplete number of correspondences compared to the full set of correspondences in the SISYPHUS alignment. This explains the linear trend in the graph. At the very end of the NCORE scale, some points are spread under the diagonal (top right corner) indicating that more peripheral or “noisy” residues are indentified in the MSA. However, the spread on the top left corner of the plot means that even when the MSA corresponds only to a small portion of the final alignment, a high score could be obtained. This illustrates the good performance of the sieving process in identifying structurally conserved residues when only small portions of the alignment are considered.

We have also compared the performance of the MUSTANG alignment under what Sippl & Wiederstein [10] refer to as “difficult structural alignments”, in the sense that structures are assigned to different SCOP or CATH folds or topologies. The difficult alignment test sets are taken from Table 1 of their publication (ADP-ribosylating toxins (SCOP folds d.166.1.1) and Poly-ADP-ribose polymerase (d.166.1.2)[10]. The results are made available as a web link to our server (see supplementary material; superposition corresponding to Case (B) being an alternative superposition of Case (A) is not produced). We found that MUSTANG was able to produce similar alignments as the authors, except in Case (F) where similarity is at a local level and numerous peripheral elements to the core (as identified by TopMatch [11]) prevented MUSTANG from recognizing and aligning the structures.

**Figure 5:**
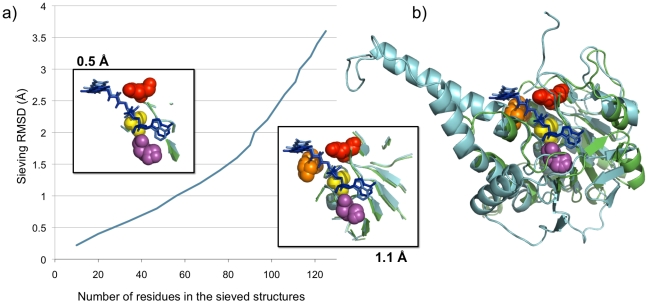
Lesk-Hubbard plot and aligned structures of d1gt8a4 and d1mo9a1. A) Plot of number of residue correspondences vs. RMSD in each structure (d1gt8a4 is in green, d1mo9a1 in cyan). Both domains exhibit an identical curve. The boxes highlight superpositions of sieved structures with their corresponding RMSD. Van der Waals representations of residues are, for d1gt8a4 and d1mo9a1 respectively: D481/D353 in red, A198/A54 in yellow, E128/E73 in magenta and T489/M361 in orange. FAD molecules are represented as sticks in dark blue (d1gt8a4) and pale blue (d1mo9a1); B) Structural alignment of the domains with bound FAD as generated by MUSTANG.

Finally, to evaluate whether the ‘sieving’ method was able to identify residues of functional relevance for “difficult” alignments, it was applied to two FAD binding domains, d1gt8a4 and d1mo9a1 (Case (D), SCOP folds c.4.1.1.1 and c.3.1.5 respectively). While MUSTANG alignment results in a 3.6 Å RMSD superposition (figure 5b), two conserved specific side-chain mediated hydrogen bonds responsible for FAD binding are identified at a sieving level as low as 0.5 Å (E218/D73 – FAD O2B/O3B; D481/D353 - FAD O3′). Conserved backbone-mediated hydrogen bonds are also identified from 0.5 Å (A198/A54 & D481/D353 bind to the phosphate group) and 1.1 Å (T489/M361 – FAD O2, figure 5a) sieved structures. Other hydrogen bonds are found specific to each domain and the way they bind FAD, they are not aligned within TopMatch and MUSTANG. This provides another example of the value of the ‘sieving’ approach in highlighting common structural features.

### Sieved structures as phase models in MR probes

To demonstrate the utility of sieved models in MR calculations (mode bias removal) we use the TTHA0727 protein from Thermus thermophilus HB8 (PDB ID 2CWQ; [12]). TTHA0727 belongs to the AhpD-like family (SCOPid 69118), with three copies of the monomer in the ASU. This structure was determined using Multiwavelength Anomalous Dispersion (MAD) phasing, since suitable homologues were not available for MR at the time. We have chosen this model because homologues now exist but are sufficiently dissimilar in sequence that they represent a challenging MR test case. The FFAS Server [13] identified a range of AhpD-like homologues with sequence identities between 16–20%. ‘Mixed’ models of the top six hits (non-conserved residues converted to alanines) were used in addition to testing a range (0.8 to 2.6 Å) of RMSD inputs within PHASER [14].

The highest resulting Z score achieved was 6.3, below the threshold of a statistically significant result. When ‘sieving’ was applied to the ‘mixed’ models however (with the same RMSD screen), numerous solutions resulted with Z scores above 7. The best of these (Z score of 10) refined well in maximum-likelihood refinement using REFMAC [15] *R*_free_ of 47% after 20 cycles) and the output built to near completion (342 residues of 411 possible) and an *R*_free_ of 25% using ARP/wARP [16]. This result indicates clearly the potential of sieving in molecular replacement.

For substructure identification, our server provides a simple method of rapidly sieving aligned structures such that conserved substructure can be identified. It also provides a basis for standardized structural comparison, as well as identification of biologically functional residues. In the case of model bias removal, testing several sieved models in a typical post-MR refinement protocol showed variability in crystallographic *R*_free_ values, which allowed models with lowest *R*_free_, and thus lowest model bias to be identified and chosen as the most promising starting models for structure refinement.

### Availability and Future Directions

Mustang-MR Sieving server is freely available at http://pxgrid.med.monash.edu.au/mustangmr-server. In the short term we are adding interface improvements in order to improve usability, as well as adding working examples of actual uses for the server. In the longer term we are working on improving various aspects of usability, specifically the ability to run several alignment jobs concurrently, with the provision of session identifiers such that users can quickly retrieve results of runs, as well as email notifications of run completion. The storage of and retrieval of previous alignments via user accounts is also planned. Regarding new functionality, the MUSTANG algorithm in general is being extended to handle non-linear (in the order of residues in the chain) multiple structural alignments and superpositions, which will automatically be merged into the server. We also aim to provide domain-refined alignment for multi-domain proteins.
